# Diagnostic reference level quantities for adult chest and abdomen-pelvis CT examinations: correlation with organ doses

**DOI:** 10.1186/s13244-023-01403-y

**Published:** 2023-04-07

**Authors:** Paulo Roberto Costa, Alessandra Tomal, Jullianna Cristina de Oliveira Castro, Isabella Paziam Fernandes Nunes, Denise Yanikian Nersissian, Márcio Valente Yamada Sawamura, Hilton Leão Filho, Choonsik Lee

**Affiliations:** 1 Institute of Physics, University of São Paulo, R. Do Matão, 1371, Butantã, São Paulo, SP 05508-090 Brazil; 2 Institute of Physics Gleb Watagin, University of Campinas, Campinas, Brazil; 3 Medical School, University of São Paulo, São Paulo, Brazil; 4 Division of Radiology, Medical School, University of São Paulo, São Paulo, Brazil; 5 Division of Cancer Epidemiology and Genetics, National Cancer Institute, National Institutes of Health (NIH), Bethesda, MD USA

**Keywords:** Organ doses, Computed tomography, Chest, Abdomen-pelvis, Statistics

## Abstract

**Objectives:**

To evaluate correlations between DRL quantities (DRLq) stratified into patient size groups for non-contrast chest and abdomen-pelvis CT examinations in adult patients and the corresponding organ doses.

**Methods:**

This study presents correlations between DRLq  (CTDI_vol_, DLP and SSDE) stratified into patient size ranges and corresponding organ doses shared in four groups: inside, peripheral, distributed and outside. The demographic, technical and dosimetric parameters were used to identify the influence of these quantities in organ doses. A robust statistical method was implemented in order to establish these correlations and its statistical significance.

**Results:**

Median values of the grouped organ doses are presented according to the effective diameter ranges. Organ doses in the regions inside the imaged area are higher than the organ doses in peripheral, distributed and outside regions, excepted to the peripheral doses associated with chest examinations. Different levels of statistical significance between organ doses and the DRLq were presented.

**Conclusions:**

Correlations between DRLq and target-organ doses associated with clinical practice can support guidance’s to the establishment of optimization criteria. SSDE demonstrated to be significant in the evaluation of organ doses is also highlighted. The proposed model allows the design of optimization actions with specific risk-reduction results.

**Supplementary Information:**

The online version contains supplementary material available at 10.1186/s13244-023-01403-y.

## Key points

1. DRL quantities and target-organ doses can support guidance’s to optimization criteria.
2. Organ doses inside imaged area are higher than in other regions.
3. Statistical significance between organ doses and the DRL quantities are presented.
4. Results reinforce the importance on the adequate choice of the scan length.

**Supplementary Information:**

The online version contains supplementary material available at 10.1186/s13244-023-01403-y.

## Background

There is an increasing concern to implement optimized CT protocols, in order to adequate the radiation dose to the patient without loss of image quality and diagnostic information [1]. The diagnostic reference level (DRL) approach was introduced decades ago [2, 3] and adopted by International Commission on Radiological Protection (ICRP) in its publication 73 [4]. A complete guidance for the DRL practical application is presented in the ICRP 135 [5]. This publication emphasizes that the DRLs must adopt measurable quantities in order to assess and compare doses for particular types of examinations among different facilities and can be used as a tool to optimize medical exposures [6–8].

On the other side, the ICRP 147 publication [9] recognizes that the best way to estimate the risk to individuals submitted to medical procedures using ionizing radiation is the adoption of organ/tissue doses and specific dose-risk models. The correlation of these risks in low dose levels as that normally used in diagnostic imaging is difficult and associated with complex uncertainties. Therefore, connections between organ doses and measurable quantities determined using controlled cohorts and their associations with technical factors normally adopted in the clinical practice may support the development of models to identify patient-specific risks. For example, retrospective correlations between DRL quantities and target-organ doses associated with some clinical practice can support guidance’s in the establishment of optimization criteria. This kind of approach is in agreement with the American Association of Physicists in Medicine (AAPM) position related to Medical Imaging Radiation Limits [10].

The correlation between organ doses and dosimetric DICOM-reported information was recently addressed by AAPM and EFOMP [11]. In the case of CT examinations, SSDE has shown a significant correlation with organ doses [12], but still requires improvement in both its interpretation as a patient radiation protection predictor and its suitability as a risk-associated estimator in clinical situations where organs are partially irradiated. Although these limitations add uncertainties to the organ dose estimations from SSDE, the combination of accurate clinically relevant information into Monte Carlo methods associated with validated SSDE calculations represents a valuable effort in the direction of associate DICOM header and Radiation Dose Structured Report (RDSR) available information with organ doses and patient-risk models [11]. The current improvements of this correlation between SSDE and patient-risk models associated with the method proposed in the present work allow to quantify the dose reduction expected to a given patient cohort when an optimized protocol will be introduced. This kind of quantification is not possible using CTDI-based only methods.

The implementation of the proposed method may allow the association of different operational parameters of a CT scanner for a given clinical task and image quality with patient organ doses and consequent risk-related information. It can impact the clinical operation if adopted as a decision-making tool for comparing protocols in a commissioning or optimization task.

This study presents correlations between DRL quantities (CTDI_vol_, DLP and SSDE) [5] stratified into adult patient size ranges for non-contrast chest and abdomen-pelvis CT examinations performed at the Institute of Radiology of the Medical School of the University of São Paulo (INRAD-HCFMUSP) and their corresponding organ doses shared in four groups associated with the imaging area: inside, peripheral, distributed and outside. The demographic, technical and dosimetric quantities were used to calculate organ doses in order to identify the influence of these quantities and doses in critical organs. A robust statistical method was implemented in order to investigate associations between these parameters.

## Methodology

### Database management, patient cohort and dose-related quantities

Data from chest and abdomen-pelvis CT examinations were retrospectively registered between January and September 2020 at INRAD-HCFMUSP. The study was approved by the HCFMUSP Institutional Review Board^1^ and informed consent was not required. The study included examinations performed on adult patients (> 18 years old) using four CT scanners: SCANNER 1 - Discovery 750 HD (GE Healthcare); SCANNER 2 - Aquilion CXL (Canon Medical Systems); SCANNER 3 and SCANNER 4 - Brilliance 64 (Philips Medical Systems).

Teamplay® platform (Siemens Healthineers) was used to select DICOM diagnostically validated image series corresponding to the cohorts of interest for the present study and their corresponding RDSR. These studies were sent to the institutional PACS (IntelliSpace PACS-Enterprise, Philips) and eFilm Workstation 3.1 (Merge Healthcare) was used to select the images of interest in each series. Patient demographic and dose data were extracted from the DICOM Header using ImageJ® (U. S. National Institutes of Health, Bethesda, Maryland, USA). Table 1 summarizes the basic acquisition and reconstruction parameters of the studied protocols. Additionally, the following demographic and technical parameters were also registered for each patient/protocol: gender, age, weight, height, voltage, pitch, collimation, TCM mode and mAs at central slice. Table 2 presents the number of collected examinations by each CT scanner and patient gender, age, weight, height and BMI, as well as the respective medians and minimum–maximum values. To be consistent with ICRP 135 [5] sample sizes for DRL evaluation, effective diameter ($d$) groups with less than 30 patients were excluded.

**Table 1:** The main acquisition parameters of the examination’s protocols evaluated for each CT scanner

	SCANNER 1		SCANNER 2		SCANNER 3		SCANNER 4
	Abdomen-pelvis	Chest	Abdomen-pelvis	Chest	Abdomen-pelvis	Chest	Chest
Protocol identification	Abdomen no- contrast	Chest routine	Abdomen no-contrast	Chest routine no-contrast	Abdomen no-contrast	Chest routine	Chest parenchyma mediastinum
Tube voltage (kV)	120; 140	120; 140	120	120	120	120	120; 140
Pitch	1.375	1; 1.375	0.828	1.250	0.829–0.984^4^	0.767; 0.797	0.828–0.922^4^
Automatic tube current modulation^1^	Auto + SmartmA	AutomA; Auto + SmartmA	SureExposure 3D	SureExposure 3D	ZDOM	ZDOM	ZDOM; ZDOM_ACS
Iterative reconstruction	SS50^2^	SS50^2^	AIDR 3D STD^3^	AIDR 3D STD^3^	–	–	–

^1^AutomA, ZDOM and ZDOM_ACS are automatic tube current modulation modes that use corrections in the longitudinal (z) direction. Auto + SmartmA and SureExposure 3D combine longitudinal (z) and angular (xy) corrections

^2^ SS50 means that 50% of the reconstruction were from iterative data

^3^Standard Iterative reconstructions (STD), which uses a standard deviation of 10 (established by the manufacture)

^4^ Pitch range

**Table 2:** Demographic distribution of collected data by CT scanner. Median and max–min values of patient´s age and weight and height are presented

		Number of examinations per scanner				Median and range (max–min) of weight, height and BMI		
Protocol	Scanner	Total	Female	Male	Median age (max–min)	Weight (kg)	Height (cm)	BMI (kg/m^2^)
Chest	SCANNER 1	158	89 (56%)	69 (44%)	56 (18–83)	70 (40–115)	175 (155–190)	23.9 (16.5–37.6)
	SCANNER 2	101	58 (57%)	43 (43%)	55 (21–86)	70 (45–125)	175 (155–190)	22.9 (16.5–36.5)
	SCANNER 3	144	83 (58%)	61 (42%)	52 (20–83)	65 (40–125)	175 (150–190)	22.9 (16.5–36.5)
	SCANNER 4	234	96 (41%)	138 (59%)	55 (18–94)	75 (35–135)	175 (155–190)	24.2 (12.9–39.4)
	Total	637	327 (51%)	312 (49%)	55 (18–94)	70 (35–135)	175 (150–190)	23.1 (12.9–39.4)
Abdomen-pelvis*	SCANNER 1	104	67 (64%)	37 (36%)	54 (21–86)	75 (40–130)	170 (150–190)	25.4 (17.8–45.0)
	SCANNER 2	103	59 (57%)	44 (43%)	51 (20–89)	75 (40–120)	170 (150–190)	24.9 (16.5–34.3)
	SCANNER 3	112	60(54%)	52 (46%)	52 (19–93)	70 (40–120)	170 (150–190)	25.1 (16.5–33.2)
	Total	319	186(58%)	133 (42%)	52 (19–93)	75 (40–130)	170 (150–190)	25.2 (16.5–45)

^*^Scanner 4 was used as emergency dedicated equipment and during the COVID’s pandemic period there wasn’t significate sample for abdomen-pelvis protocols

This study adopted the CTDI_vol_ and DLP values presented in the RDSR. The reference phantom size for all CTDI_vol_ data was 32 cm. SSDE was estimated according to the AAPM Report No. 204 [14] as the product of the CTDI_vol_ and a size-dependent factor. The uncertainties associated with each evaluated quantity were estimated by considering the accuracy presented on the RDSR, and the typical uncertainties estimated during quality control measurements. The maximum CTDI_vol_ and DLP uncertainties were, respectively, 4.1%, and 1.5%, considering a 95% confidence level (*k* = 2). A 20% uncertainty was associated with the quantity SSDE [13]. The effective diameter, $d$, was chosen as patient size discriminator [14] and the body mass index (BMI) was alternatively tested on the statistical analysis.

### Organ dose estimations

NCICT 2.0 software [13–17] was used to estimate doses in target-organs. This software has the capability to calculate body size-dependent organ doses using computational phantoms and it takes into consideration both the patient´s anatomy (weight and height) and scanner/protocol-specific characteristics (kV, mAs, pitch, collimation, TCM strength, etc.). The incorporation of these variabilities allows to associate these characteristics on the cohort´s organ dose responses.

In the present work, the organs were grouped adopting Li et al. [18] classification considering chest and abdomen-pelvis scans:

#### Chest scans

- Inside organs (IO): lungs, breasts, heart, and thymus
- Peripheral organs (PO): thyroid, esophagus, liver, gall bladder, stomach, spleen, pancreas, adrenal glands, colon, rectosigmoid, and small intestine
- Distributed organs (DO): Active marrow, shallow marrow, spinal cord, skin, and residual soft tissues
- Outside organs (OO): brain, salivary glands, oral cavity, pituitary glands, larynx-pharynx, kidneys, bladder, ovaries, and uterus.

#### Abdomen-pelvis scans

- Inside organs (IO): liver, gall bladder, stomach, spleen, pancreas, adrenal glands, kidneys, colon, rectosigmoid, small intestine, urinary bladder, ovaries, and uterus
- Peripheral organs (PO): lungs, breasts, esophagus, and heart
- Distributed organs (DO): Active marrow, shallow marrow, spinal cord, and skin
- Outside organs (OO): brain, thyroid, salivary glands, oral cavity, pituitary glands, and thymus.

This classification is convenient since allows to compare the relative influence of the x-ray beam incidence on the irradiated organs considering different anatomic regions (chest or abdomen-pelvis). According to Li et al. [18], it is expected that inside and peripheral organs may account for around 90% of the effective dose associated with chest and abdomen-pelvis CT scans. Another advantage of this classification is that it facilitates the identification or inadequate use of the scan length, since it will be reflected on the doses in regions out of the diagnostic interest volume, in special on peripheral organs group.

Considering $D_{k}$ the dose in the *k*th organ or tissue into a group with *M* components and $w_{k}$ its respective ICRP 103 [19] tissue weighting factor, the Organ Dose Group (ODG) contributions of each organ group (IO, PO, DO or PO) to the total patient´s effective dose were calculated as:

1 $${\text{ODG}} = \mathop \sum \limits_{k = 1}^{M} w_{k} D_{k}$$

The anatomical and demographical information from all patients of the cohort as well as technical data corresponding to the CT equipment and protocol (manufacturer and model, voltage, TCM strength, pitch, collimation, scan length and CTDI_vol_) for each examination were used as input to the NCICT software. Therefore, the NCICT´s organ doses are representative of the distribution of the organ doses in the evaluated cohorts. This classification was adopted considering each body region (chest or abdomen-pelvis). The correlations regarding these data and the organ dose groups for chest and abdomen-pelvis anatomical regions were evaluated.

### Variation of organ doses groups with SSDE and effective diameter

In order to establish simplified functional relations between the organ doses groups and the corresponding SSDEs and effective diameters, $d$, the following power functions were adopted:

2 $${\text{ODG}} = \alpha \times {\text{SSDE}}^{\beta } \;{\text{or}}\;{\text{ODG}} = \gamma \times d^{\delta }$$

In Eq. (2), ${\text{ODG}}$ represents the organ dose groups described in Eq. (1). The parameters $\alpha$, $\beta$, $\gamma$, and $\delta$ were obtained by fitting the data corresponding to the chest and abdomen-pelvis cohorts adopting the Levenberg-Maquardt method using Origin® 2020 (OriginLab Corporation). Values of these fitting parameters were obtained considering all patients grouped into the two cohorts (chest and abdomen-pelvis).

### Statistical analyses

The Kolmogorov–Smirnov test [20, 21] was implemented to evaluate the normality of distributions of the continuous variables IO, PO, DO and OO doses and their corresponding log-transformed distributions. For each evaluated scanner, the original distributions of these variables differ from normal but their log-transformed distributions can be considered normal (*p* < 0.001) [21].

Therefore, the Generalized Additive Model (GAM) [22, 23] was implemented in order to correlate the log-transformed organ dose groups (IO, PO, DO and OO) as outcomes to the scalar variables CTDI_vol_, DLP and SSDE as key predictors. The adopted covariates were $d$ (or *BMI*, alternatively), individual patient identification number and scanner identification. The detailed description and results of the GAM model are presented on the Additional file 1. All statistical analyses were performed using R Software version 4.1.2 (The R Foundation for Statistical Computing). *P* < 0.05 was considered to represent statistical significance.

## Results

### Comparative organ dose estimations

**Fig. 1:**
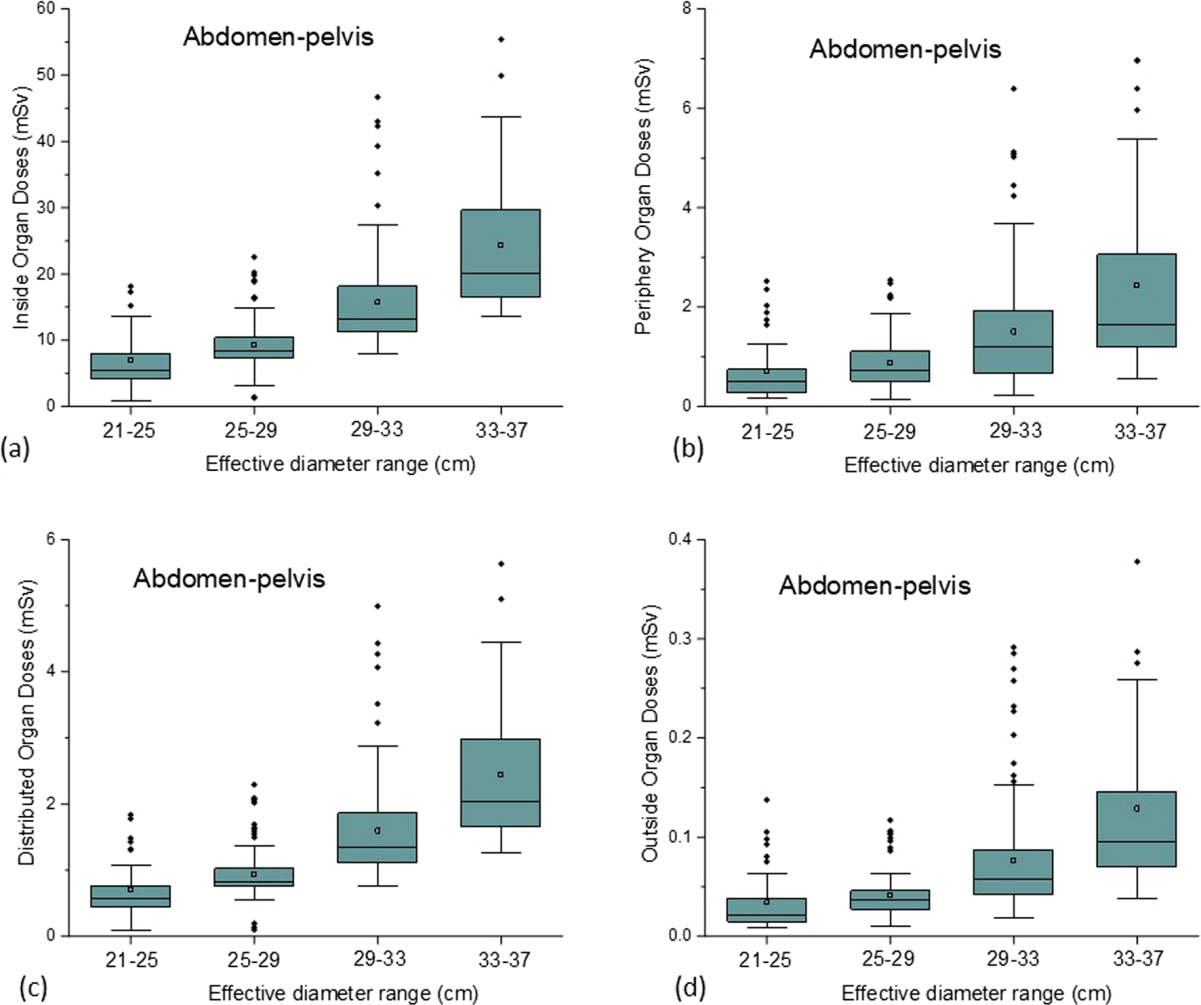
Box-plots representing the distributions of the organ dose groups: **a** IO, **b** PO, **c** DO and **d** OO, according to the effective diameter group ranges for abdomen-pelvis examinations

**Fig. 2:**
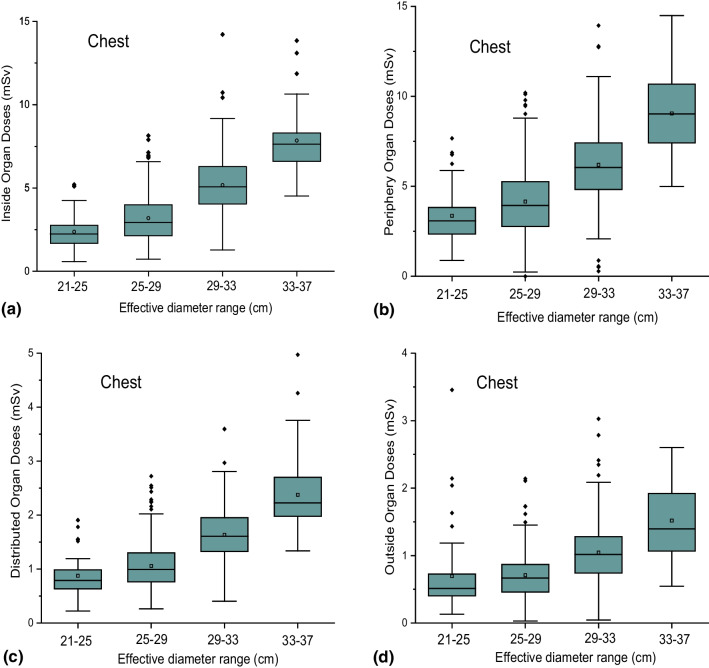
Box-plots representing the distributions of the organ dose groups: **a** IO, **b** PO, **c** DO and **d** OO, according to the effective diameter group ranges for chest examinations

Table 3 presents the median values of the organ doses grouped according to Li et al. [18] classification and the corresponding interquartile intervals [IQ1–IQ3] for chest and abdomen-pelvis examinations. The values were grouped according to *d* ranges as defined in the Methodology section. As expected, the organ doses in the regions inside the imaged area are higher than the organ doses in peripheral, distributed and outside regions. The IO doses for abdomen-pelvis examinations are around 9–10 times higher than the organ doses in other body regions. For chest examinations, the IO doses are about 7–9 times higher than that in the DO and OO doses. The exception are the PO doses associated with chest examinations, which demonstrated to have the same order of magnitude as IO doses. Figures 1 and 2 show the box-plots representations of the distributions of the organ dose groups according to the *d* ranges for abdomen-pelvis and chest examinations, respectively.

**Table 3:** Median values of the grouped organ doses and corresponding interquartile intervals for chest and abdomen-pelvis examinations. *N* represents the number of patients in the cohorts. The values are grouped according to the effective diameter ranges

			Median organ doses [IQ1–IQ3] (mSv)			
Protocol	*d* (cm)	N	Inside	Peripheral	Distributed	Outside
Chest	21–25	49	2.2 [1.7–2.8]	3.1 [2.3–3.8]	0.6 [0.2–1.0]	0.5 [0.4–0.7]
	25–29	232	2.9 [2.1–4.0]	3.9 [2.8–5.3]	1.0 [0.8–1.3]	0.7 [0.5–0.9]
	29–33	295	5.1 [4.0–6.3]	6.0 [4.8–7.4]	1.6 [1.3–2.0]	1.0 [0.7–1.3]
	33–37	57	7.6 [6.6–8.3]	9.0 [7.4–10.7]	2.2 [2.0–2.7]	1.1 [1.1–1.9]
Abdomen-pelvis	21–25	39	5.5 [4.3–8.1]	0.5 [0.3–0.78]	0.6 [0.4–0.8]	0.02 [0.01–0.03]
	25–29	111	8.4 [7.4–10.5]	0.8 [0.5–1.1]	0.8 [0.8–1.0]	0.04 [0.03–0.05]
	29–33	109	13.3 [11.3–18.2]	1.2 [0.7–1.9]	1.4 [1.1–1.9]	0.06 [0.04–0.07]
	33–37	39	20.2 [16.6–27.7]	1.7 [1.2–3.1]	2.1 [1.7–3.0]	0.09 [0.07–0.12]

**Fig. 3:**
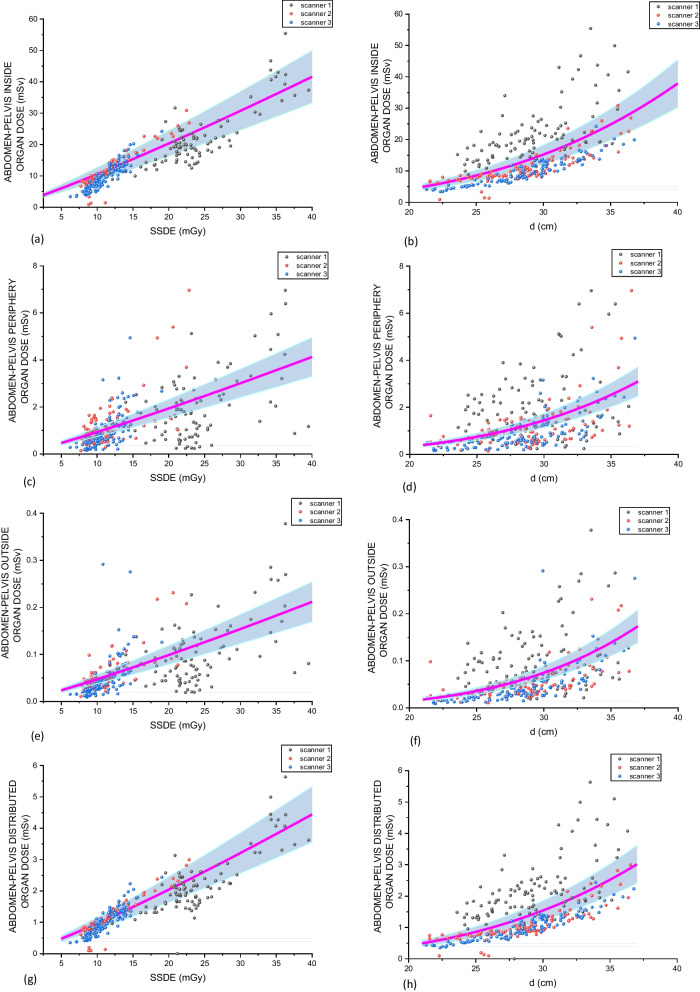
Abdomen-pelvis organ dose groups as functions of the SSDE ((**a**) Inside, (**c**) Periphery, (**e**) Outside and (**g**) Distributed) and *d * ((**b**) Inside, (**d**) Periphery, (**f**) Outside and (**h**) Distributed). The magenta lines correspond to the fittings using Eq. (2) considering the contributions of patients imaged in all CT scanners. The blue band refers to a range of ± 20% around this average line. The dots identify each CT scanner used in the study

**Fig. 4:**
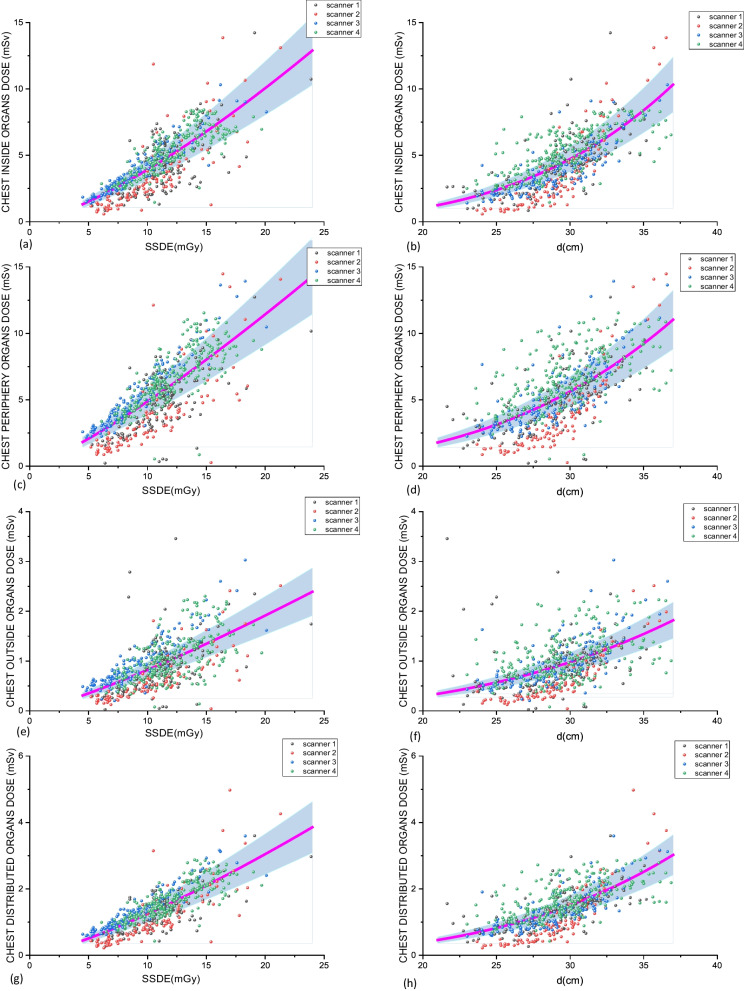
Chest organ dose groups as functions of the SSDE ((**a**) Inside, (**c**) Periphery, (**e**) Outside and (**g**) Distributed) and *d* ((**b**) Inside, (**d**) Periphery, (**f**) Outside and (**h**) Distributed). The magenta lines correspond to the fittings using Eq. 2 considering the contributions of patients imaged in all CT scanners. The blue band refers to a range of ± 20% around this average line. The dots identify each CT scanner used in the study

Table 4 presents the results of the fitting parameters of Eq. (2). These fitting parameters were assessed by grouping all patients of each cohort. The functional correlation using the adopted power law for SSDE and *d* for inside, peripheral, distributed and outside organs dose groups are shown. Figures 3 and 4 present, respectively, the abdomen-pelvis and chest organ doses group resulting fits and the distribution of the calculated organ dose groups for each patient on the cohorts and CT scanners. The blue bands correspond to ± 20% around the fitted curves.

**Table 4:** Fitting parameters of the power laws ${\text{ODG}} = {\upalpha } \times {\text{SSDE}}^{{\upbeta }}$ and ${\text{ODG}} = {\upgamma } \times {\text{d}}^{{\updelta }}$. These fitting parameters were assessed grouping all patients of each cohort. The functional correlation using the adopted power law for SSDE and d for inside, peripheral, distributed and outside organs dose groups are shown. The fitting parameters were calculated using the Levenberg–Maquardt Method incorporated in the software Origin® 2020

		$\mathbf{O}\mathbf{D}\mathbf{G}=\boldsymbol{\alpha }\times {\mathbf{S}\mathbf{S}\mathbf{D}\mathbf{E}}^{{\varvec{\beta}}}$					$\mathbf{O}\mathbf{D}\mathbf{G}={\varvec{\gamma}}\times {{\varvec{d}}}^{{\varvec{\delta}}}$			
		Inside	Peripheral	Distributed	Outside		Inside	Peripheral	Distributed	Outside
Abdomen-pelvis	*α*	6.25×10^-1^	6.21×10^-2^	5.98×10^-2^	3.07×10^-3^	*γ*	3.16×10^-4^	6.29×10^-6^	2.89×10^-5^	8.61×10^-8^
	*β*	1.14	1.13	1.16	1.14	*δ*	3.17	3.63	3.20	4.02
Chest	*α*	1.71×10^-1^	2.87×10^-1^	6.39×10^-2^	4.95×10^-2^	*γ*	1.46×10^-5^	1.02×10^-4^	1.88×10^-5^	4.30×10^-5^
	*β*	1.36	1.23	1.29	1.22	*δ*	3.73	3.21	3.32	2.95

The relation between ODGs and SSDE is approximately linear, with 1.13 ≤ $\beta$ ≤ 1.16 for abdomen-pelvis examinations and 1.22 ≤ $\beta$ ≤ 1.36 for chest procedures. A stronger influence on ODGs was found considering *d*. In this case, the $\delta$ parameter variated between 3.20 and 4.02 for abdomen-pelvis examinations and 2.95 and 3.73 for chest examinations. Additionally, the $\alpha$ and $\gamma$ fitting parameters may provide information regarding the magnitude of the doses in both procedures and organ dose groups. Observing these parameters, it can be noted that the IO doses for abdomen-pelvis are one or two order of magnitudes higher than doses for other organ groups for a same SSDE or effective diameter. However, the $\alpha$ parameter for IO and PO doses are approximately similar in chest examinations, but this parameter is about ten times lower for IO doses than for PO doses in abdomen-pelvis examinations. These kinds of finding will be discussed in the next section.

### Statistical evaluation

The application of the GAM presented in the Methodology section for each cohort of patients and anatomical region allowed the identification of levels of significance of each key predictor (CTDI_vol_, DLP and SSDE) on the resulting organ doses (outcomes). The results allow to identify which of these scalar variables are significant or not on the resulting organ doses adopting a significance level of 0.05. Additional file 1 presents the p-values associating each key predictor with the studied organ dose groups as well as plots associating the distribution of the input data and the corresponding model responses.

For IO and DO doses of chest examinations, the model identified statistical significance on the DLP and SSDE, while for PO and OO doses, CTDI_vol_ and DLP were demonstrated to be statistically significant. All CT scanners were demonstrated to be significant in the estimation of additive function for all organ dose groups to account to the effective diameter, *d*, except scanner 4 which is not significant for estimating PO doses, and scanner 3 and scanner 4, which are not significant to estimating OO doses. In this part of the application of the model, its resulting accuracy ($R^{2}$) was estimated as 0.844, 0.662, 0.822 and 0.609 for fitting, respectively, the IO, PO, DO and OO dose groups considering the adopted key predictors and covariates. The variation explained by the model ranged between 62 and 85%. The alternative evaluation replacing *d* by BMI as a key predictor in the chest cohort resulted in similar levels of significance of all variables. However, in this case, only DLP was statistically significant to all organ group doses, CTDI_vol_ is not significant to PO doses and SSDE is only significant for IO doses. In this case, scanner 3 and scanner 4, are only significant for estimating IO doses. The accuracy of this alternative implementation was estimated as 0.877, 0.673, 0.828 and 0.605 for fitting respectively the IO, PO, DO and OO dose groups. The deviance explained by the model ranged from 61 to 88%.

The application of GAM to the abdomen-pelvis cohort demonstrates statistical significance only of SSDE for modeling patients´ IO and DO doses. Additionally, all CT scanners are significant to estimate the IO doses, while scanner 1 was not significant to estimate the DO doses. DLP was significant to estimate PO and OO doses and only scanner 2 was not significant to estimate these groups of organ doses. CTDI_vol_ was also significant to PO dose estimations. In this case, the model´s accuracies were estimated as 0.846, 0.541, 0.842 and 0.664 for fitting respectively the IO, PO, DO and OO dose groups. The deviance explained by the model ranged between 55 and 85%. The adoption of the alternative implementation of the GAM considering the BMI replacing *d* resulted in similar levels of significance for all key predictors. However, CTDI_vol_ is significant for IO, PO and OO doses. DLP is only significant for PO and OO doses. SSDE and all scanners are significant in estimating all groups’ organ doses. The accuracies were estimated as 0.856, 0.689, 0.858, and 0.8 for fitting respectively the IO, PO, DO and OO dose groups. The deviance explained by the model ranged between 71 and 86%.

## Discussion

The evaluation of the functional correlations between the organ dose groups and the SSDE and *d* allows to define the impact of these variables on critical organ doses and also estimate doses in these organ groups taking into account these variables. Table 4 provides the fitting parameters of the power law presented in Eq. (2) and Figs. 3 and 4 show these functional behaviors and the real cohorts data.

The fact that the doses in chest PO presented the same order of magnitude of the IO doses can be accounted for by the fact that organs such as stomach, spleen, pancreas and gall bladder, which are close to the volume of interest in chest examinations and has high ICRP 103 weighting factors values (0.12), are probably irradiated during routine chest imaging. Additionally, the esophagus is considered a PO in chest examinations according to the Li et al. [18] classification, but part of this organ is usually inside the patient volume irradiated by the primary beam in this kind of imaging procedure. This effect of the esophagus, liver, gall bladder, stomach and spleen in PO doses can also be seen in Li et al. [18] results, where the authors present plots of organ dose conversion factors as a function of modulation control strength.

In the case of the ODGs distributions associated with abdomen-pelvis examinations (Fig. 3), it can be noted a huge dispersion of the data around the fitted curve, in special considering the distributions as functions of the effective diameters. In these cases, scanner 1 demonstrates more dispersion and higher doses them scanners 2 and 3 for all ODGs. The distributions associated with SSDE are less dispersive, but the higher doses associated with scanner 1 can also be observed on the larger SSDE values in comparison with scanners 2 and 3. It can also be noted that, for abdomen, the IO doses are around 10 times higher than the DO and PO doses and more than 100 times higher than OO doses.

Considering the chest ODG distributions (Fig. 4), the dispersions are not so evident as that resulted from abdomen-pelvis data. In these cases, doses associated with all four scanners distribute quite equally around the fitted curve, in special that correlated to effective diameter. However, it can be noted that scanners 2 and 3 present dose distributions below the fitted curve while scanner 4 presents higher doses considering all dose groups. Finally, in this case, the PO doses are in the same order of magnitude of the IO doses and both are 2–3 times higher than OO and DO doses.

These variations on the fit parameters and consequently on the organ doses may also be associated with the adopted protocols in each evaluated scanner. Abdomen-pelvis protocols in scanner 1 are implemented using pitches higher than scanners 2 and 3. The reflection of these protocols’ differences may be associated with a slightly higher IO dose associated with scanners 2 and 3 in comparison to scanner 1. The same association can be done considering chest studies, where scanners 1 and 2 use higher pitches than scanners 3 and 4. The higher doses in abdomen-pelvis examinations associated with scanner 1 independently of the effective diameter of the patients may also be associated with the use of both 120 kV and 140 kV in these procedures.

The evaluation of the deviance explained resulting from the application of the GAM can be grouped in two ranges: [55%; 81%] for PO and OO doses and [83%; 88%] for IO and DO doses. The good accuracy of the model for estimating IO and DO doses is associated with both the adequacy of anatomical regions really irradiated during the scan procedures and the effective influence of the CT x-ray beam on the distributed organs around the patient’s body. However, as it is usual on application of GAM in medical [24, 25] or non-medical areas [26], some low values of the deviance explained calculated by the model may indicate the need of inclusion of additional covariates into de modeling. In the present study, the lower values of deviance explained associated with PO doses, independently of the adoption of *d* or BMI as key predictor, may indicate that the organ classification proposed by Li et al. [18] is not well correlated to the real organ distribution associated with the examinations or the adoption of scanner lengths over the expected values.

It was observed that the significance of CT scanners depends on the anatomic region examined (chest or abdomen-pelvis), patient size discriminator (*d* or BMI) and OD group (IO, PO, DO or OO). For chest, only scanners 1 and 2 were significant on SDDE estimation for all cases, and for abdomen-pelvis examinations only scanner 3. Additionally, for chest examinations, Fig. 4 demonstrates that the model is scanner-sensitive, since can be noted that scanners 1 and 2 are associated with lower organ doses than the other scanners. It is not so evident considering abdomen-pelvis examinations (Fig. 3). This fact also demonstrates the sensitivity of the model to the exposed body-region.

The present work used retrospective adult patient databases of typical CT abdomen-pelvis and chest examinations in scanners from three different vendors. Although the patient samples and machine technologies evaluated be representative of a very popular clinical situation, the statistical results must be carefully interpreted and not generalized. The study can be understood as a proof-of-concept that the systematic combination of protocol-related information, RSDR results, patient demography and an organ-dose estimation tool may help radiologists and medical physicists on the decision-making process during the optimization of a given clinical task. For example, by observing Fig. 3, the institutional team dealing with protocol optimization can note that scanner 1 is responsible for higher organ doses than scanners 2 and 3 in abdomen-pelvis examinations and these higher doses are not correlated to oversized patients, since the effective diameter range for the three scanners is the same. Table 1 shows that this scanner uses pitch and voltages higher than the other two. If there are no significant differences in image quality that justify these higher doses, this would be the priority of the team on the abdomen-pelvis protocol examinations. The quite similar optimization process but considering chest examinations can be addressed using Fig. 4. In this case, it can be noted that scanner 2 represents the lower organ doses and scanner 4 the higher organ doses. Table 1 shows that scanner 2 uses higher pitch values than scanner 4. Additionally, scanner 4 adopts both 120 and 140 kV and does not use iterative reconstruction. Taking into account just these facts, some optimization options may be suggested by the local team in order to drive scanner 4 to lower doses. However, it must be done with care, since this scanner works as an emergency dedicated equipment in the hospital, which is a regional reference center that received chronic patients during the pandemic period included in the data collection. Therefore, in some special cases, higher doses must be justified by the clinical needs.

Future improvements considering clinical indications [6, 28] may also improve the applicability of the method. This kind of approach is intrinsically limited by the reduced number of machines and protocols included in the real-data evaluation. In particular, the large variability of TCM strategies and local adoption of different iterative reconstruction algorithms will always result in machine-cohort-protocol dependence. However, these limitations do not reduce the applicability of the method to other clinically relevant situations. Finally, special care must be taken when one intends to analyze the results in terms of organ dose groups, since in some cases the partial irradiation may introduce bias, as previously discussed in the case of the esophagus, considered a peripheral organ in chest examinations.

## Conclusion

Inside and periphery organ doses represent more than 90% of the total doses in both studied cohorts, in agreement with Li et al. [18]. As expected, organ doses inside the diagnostic region-of-interest are systematically higher than other regions, except for periphery organs during chest examinations. It reinforces the importance on the adequate choice of the scan length. In this case, unnecessary incidence of the primary x-ray beam on periphery organs which are not required for clinical evaluation may impact on the collective doses from chest patient cohorts. This fact is also reinforced by the statistical evaluation. As DLP depends on the scan length, the correct adjustment of this parameter by the technologists is essential for effective dose optimization.

The significance of SSDE in the evaluation of organ doses is also highlighted. Previous works [11, 12, 27, 28] demonstrated that this quantity is correlated with patient organ doses. Our results demonstrated strong statistical correlation associating SSDE with inside and distributed organ doses in the chest and abdomen-pelvis cohorts and it was also statistically significant on the periphery and outside organ doses in the last case. Important effort of the scientific community has been done in order to emphasize the association of this quantity with adult [29, 30] and pediatric [31–34] patient doses.

Therefore, the modeling and results of the present work may contribute to the development of more accurate dose-risk models [9] associated with DRL quantities. The importance of the correlation between SSDE and organ doses can be emphasized by the possibility of developing analytical tools for clinical protocols commissioning process, associating risk-related data with image quality parameters. However, special care must be taken in this kind of association given its intrinsic complexity. A recent work from Ria et al. [35] highlighted the caveats in making decisions associated with clinical practices considering “implicit risk to factors that do not closely reflect risk.” The authors did a very careful and interesting study associating a clinical dataset of chest and abdominal-pelvic protocols in order to compare how different radiation risk surrogates characterize radiation burden. In total, the authors evaluated twelve proposed or in-use risk surrogates and their burden attributes using linear regression models. They demonstrated the advantages and limitations of using each one, in special the low sensitivity of the SSDE as a patient risk metric. A second recent contribution in this field was published by Zewde et al. [36]. The authors estimated cumulative organ doses and age- and gender-stratified cancer mortality risks in patients undergoing recurrent CT examinations. Adopting a retrospective study in a large database, the authors’ findings demonstrated consistency that organ doses are the best quantity to assess radiation risks, emphasizing that risk estimations must be interpreted as average estimations applicable to patient groups and not for individuals. These works adopted different Monte Carlo methods in order to associate technical CT parameters and patient anatomies to organ doses and are examples of milestones in the association of these data to patient risk. It is clear the necessity of additional efforts of the scientific community in both availability of more consistent organ dose-risk data and its careful interpretations before these correlations can be fully adopted for CT protocol optimization. Our work is a clear contribution to these developments since the progress and applicability of these patient-specific methods require a strong association with practical scenarios and resulting organ doses.

Using the presented methodology, CT image chain stakeholders in a radiology department can identify and prioritize what scanner-specific and/or procedures-specific technical parameters may be focused in order to conduct an effective optimization process. Additionally, the connection between DRL quantities, patient demographic information and organ doses allows the association of these optimization actions with specific risk-reduction results.

## Supplementary Information

**Additional file 1: **Statistical evaluation.

## Data Availability

The datasets used and/or analyzed during the current study are available from the corresponding author on reasonable request.

## Abbreviations

AAPM: American Association of Physicists in Medicine
BMI: Body mass index
CTDI_vol_: Volume CT dose index
DLP: Dose-length product
DO: Distributed organs
DRL: Diagnostic reference level
DRLq: DRL quantities (CTDI_vol_, DLP and SSDE)
GAM: Generalized additive model
ICRP: International Commission on Radiological Protection
IO: Inside organs
INRAD-HCFMUSP: Institute of Radiology of the Medical School of the University of São Paulo
OO: Outside organs
PO: Peripheral organs
RDSR: Radiation dose structured report
SSDE: Size-specific dose estimate
TCM: Tube current modulation

## Footnotes

1 Ethics Committee approval CAAE 27,912,619.6.0000.0068.
