# Supplementary material for: Diagnostic reference level quantities for adult chest and abdomen-pelvis CT examinations: correlation with organ doses
Source: Insights Imaging. 2023 Apr 7;14:60. doi: 10.1186/s13244-023-01403-y (PMC10079797; doi:10.1186/s13244-023-01403-y)
Supplement: Supplementary file 1 — Additional file 1: Statistical evaluation. [file 13244_2023_1403_MOESM1_ESM.pdf]

SUPPLEMENTAL MATERIAL – STATISTICAL EVALUATION

Diagnostic Reference Level quantities for adult chest and abdomen-pelvis CT examinations: correlation with organ doses

Methods

The Generalized Additive Model (GAM) (1, 2) was implemented in order to correlate the log-transformed of the organ dose groups (inside organs - IO, peripheral organs - PO, distributed organs - DO and outside organs - OO) as outcomes to the multiple scalar variables $CTDI_{vol}$, DLP and SSDE. The adopted covariates were effective diameter d (or body mass index, *BMI*, alternatively), individual patient identification number and the scanner identification as key predictors

GAM allows to accommodate functional variables, $f(\cdot)$, which uses a set of smoothing functions, one for each CT machine, to account to the d_i values. Therefore, considering μ_i as the logarithm of each organ dose group of the ith examination, the model can be described as

$$\mu_i = \beta_0 + \sum_{j=1}^{3} \beta_j F_{i,j} + f(d_i) \quad (1)$$

Where

$$f(d_i) = \sum_{j=1}^{N} g_j(d_i) G_{i,j} \quad (2)$$

In equation (1), $F_{i,j}$ represents the variables $CTDI_{vol}$ ($j = 1$), DLP ($j = 2$) and SSDE ($j = 3$) associated to the ith examination. In addition, in equation (2), *N* is the number of CT machines (4 and 3 for chest and abdomen protocols, respectively), $G_{i,j}$ is a binary variable which is equal to 1 when the CT machine used in the examination the ith patient is the CT_j, where $j = 1 \cdots N$, and it is 0 otherwise.

Finally, the values β_0 and β_j ($j = 1,2$ and 3) are parameters to be estimated by the model and $g_j(d_i)$ are functions which can be estimated by a linear combination of B-spline functions, represented by

$$g_j(d_i) = \sum_{k=1}^{K} b_k(d_i) \theta_{jk} \quad (3)$$

In this case, K is the number of cubic spline functions, b_k are the kth base functions of the applied B-splines on a given observed d_i, and θ_{jk} are the parameters of each base-function b_k. An alternative evaluation was also implemented replacing d by the BMI in order to evaluate the response of the model using this variable.

The GAM model presented in equation (1) was fitted by adjusting the parameters β and θ, using the Penalized Iteratively Reweighted Least Squares (P-IRLS) method. The accuracy of the model was evaluated considering the determination coefficient (3), R^2, which allows the estimation of the proportion of the data variability resulting to the fitting model. All statistical analyses were conducted in R Software version 4.1.2 (The R Foundation for Statistical Computing, Austria). A p-value less than 0.05 was considered to represent statistical significance.

Results

Tables S1 and S2 show the significance levels (p-value) of each scalar variable adopted at the applied GAM considering that the functional variables $f(\cdot)$ accommodate, respectively, the effective diameter values (d) and body mass index (BMI). A value of $p < 0.05$ can be considered a statistically significant contribution of the variable on each organ dose group.

Examples of results extracted from GAM for the Organ Dose groups: Inside Organs, Peripheral Organ, Distributed Organ and Outside Organ for Chest protocol are presented in Figures S1 and S2, and for Abdomen-pelvis protocol in Figures S3 and S4, considering respectively the effective diameter and body mass index as functional variables. In these figures each original Organ Dose quantity is compared to the respective GAM predictions as a function of effective diameters or body mass index, classified according the patient gender (male, M, or female, F).

Table S1 – Significance levels (p-value) of each scalar variable adopted at the applied GAM, considering the functional variables, $f(\cdot)$, as a function of the effective diameter values (d). A value of $p < 0.05$ can be considered a statistically significant contribution of the variable on each Organ Dose group.

Protocol	Scalar variables	Significance levels (p-value)								
		$CTDI_{vol}$	DLP	SSDE	SCANNER				R^2	Dev Exp
					1	2	3	4		
Chest	IO dose	0.641137	0.002537	0.000853	$< 2\times10^{-16}$	$< 2\times10^{-16}$	1.57×10^{-6}	8.01×10^{-6}	0.844	85%
	PO Dose	0.0289	7.84×10^{-16}	0.0833	$< 2\times10^{-16}$	$< 2\times10^{-16}$	0.0376	0.0504	0.662	68%
	DO dose	0.9000	9.62×10^{-12}	0.0229	$< 2\times10^{-16}$	$< 2\times10^{-16}$	0.00619	0.01162	0.822	83%
	OO dose	0.000327	$< 2\times10^{-16}$	0.553887	3.43×10^{-6}	$< 2\times10^{-16}$	0.214	0.591	0.609	62%
Abdomen-pelvis	IO dose	0.6683	0.0986	2.69×10^{-5}	0.0157	$< 2\times10^{-16}$	$< 2\times10^{-16}$	-	0.846	85%
	PO Dose	0.0406	7.52×10^{-13}	0.6339	0.02674	0.36188	0.00066	-	0.541	55%
	DO dose	0.207971	0.629663	0.000113	0.0601	$< 2\times10^{-16}$	$< 2\times10^{-16}$	-	0.842	85%
	OO dose	0.335	9.12×10^{-11}	0.535	0.0453	0.1172	1.4×10^{-5}	-	0.664	67%

Table S2 – Significance levels (p-value) of each scalar variable adopted at the applied GAM, considering the functional variables, $f(\cdot)$, as a function of the body mass index (BMI). A value of $p < 0.05$ can be considered a statistically significant contribution of the variable on each Organ Dose group.

Protocol	Scalar variables	Significance levels (p-value)								
		$CTDI_{vol}$	DLP	SSDE	SCANNER				R^2	Dev Exp
					1	2	3	4		
Chest	IO dose	0.011419	0.040440	0.000248	$< 2\times10^{-16}$	$< 2\times10^{-16}$	$< 2\times10^{-16}$	$< 2\times10^{-16}$	0.877	88%
	PO Dose	0.176	1.45×10^{-15}	0.328	$< 2\times10^{-16}$	$< 2\times10^{-16}$	0.191	0.124	0.673	68%
	DO dose	0.00019	6.42×10^{-11}	0.582024	$< 2\times10^{-16}$	$< 2\times10^{-16}$	0.0555	0.6264	0.828	83%
	OO dose	0.00058	$< 2\times10^{-16}$	0.55286	2.34×10^{-6}	$< 2\times10^{-16}$	0.271	0.352	0.605	61%
Abdomen-pelvis	IO dose	0.0284	0.3987	8.38×10^{-5}	$< 2\times10^{-16}$	$< 2\times10^{-16}$	$< 2\times10^{-16}$	-	0.856	86%
	PO Dose	4.95×10^{-16}	$< 2\times10^{-16}$	0.03	$< 2\times10^{-16}$	3.15×10^{-6}	$< 2\times10^{-16}$	-	0.689	71%
	DO dose	0.982	0.190	6.28×10^{-7}	$< 2\times10^{-16}$	$< 2\times10^{-16}$	$< 2\times10^{-16}$	-	0.858	86.%
	OO dose	5.74×10^{-16}	$< 2\times10^{-16}$	0.00325	$< 2\times10^{-16}$	$< 2\times10^{-16}$	$< 2\times10^{-16}$	-	0.800	81%

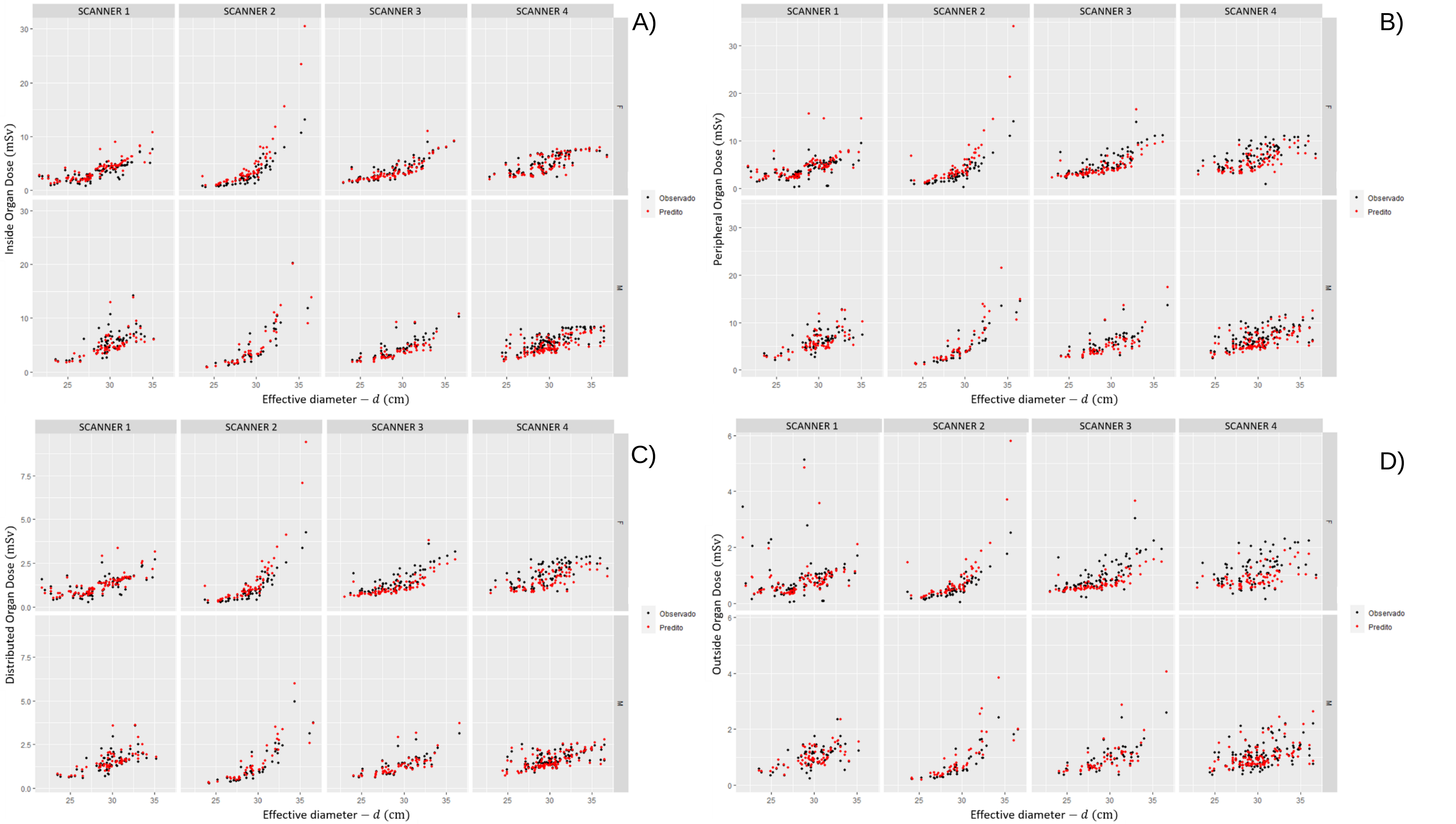

Figure S1. A) Inside Organ Dose; B) Peripheral Organ Dose; C) Distributed Organ Dose and D) Outside Organ Dose values for Chest protocol of female (F) and male (M) patients computed using NCICT (original) and predicted from GAM as a function of effective diameter, d.

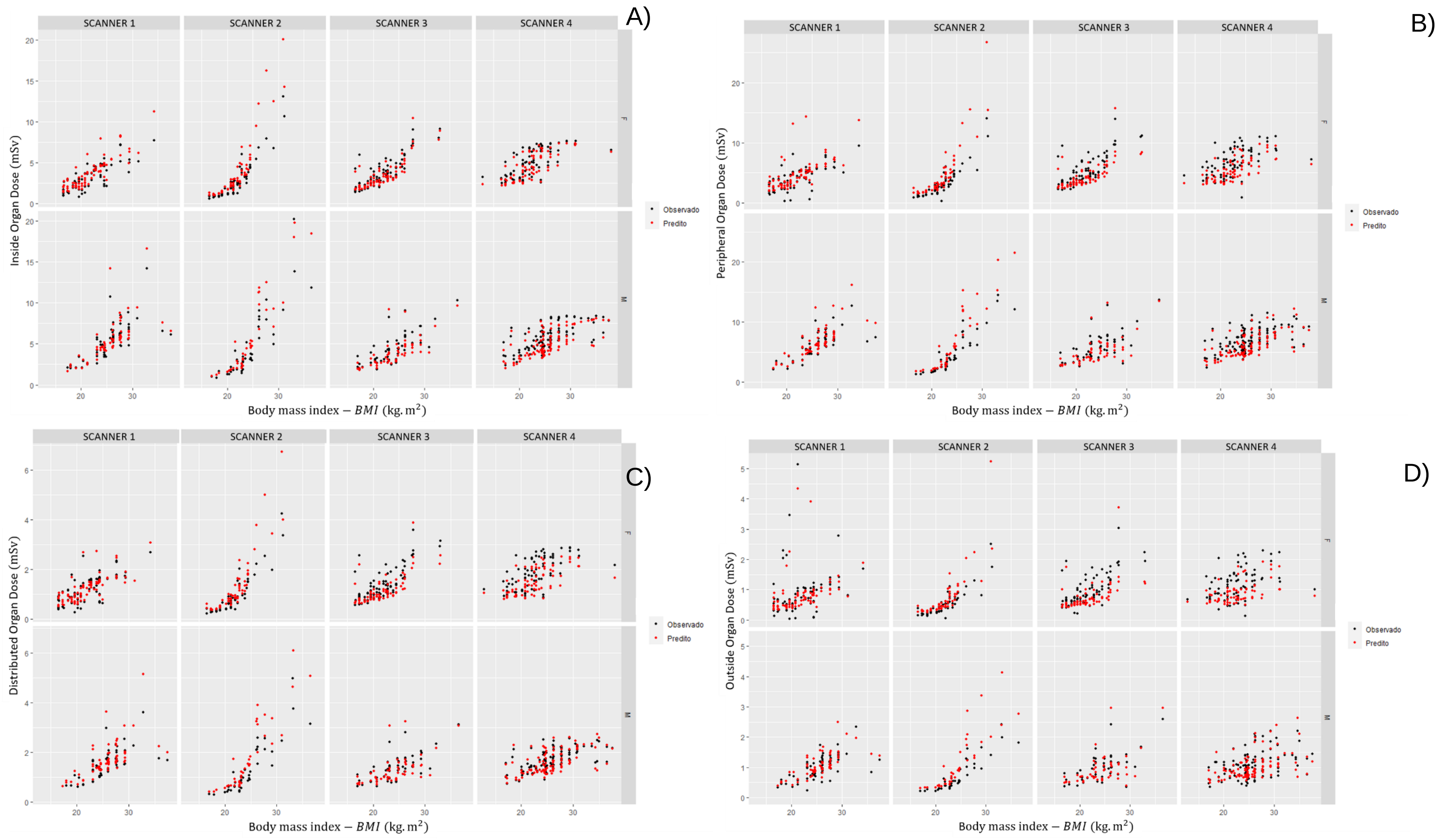

Figure S2. A) Inside Organ Dose; B) Peripheral Organ Dose; C) Distributed Organ Dose and D) Outside Organ Dose values for Chest protocol of female (F) and male (M patients computed using NCICT (original) and predicted from GAM as a function of body mass index, BMI.

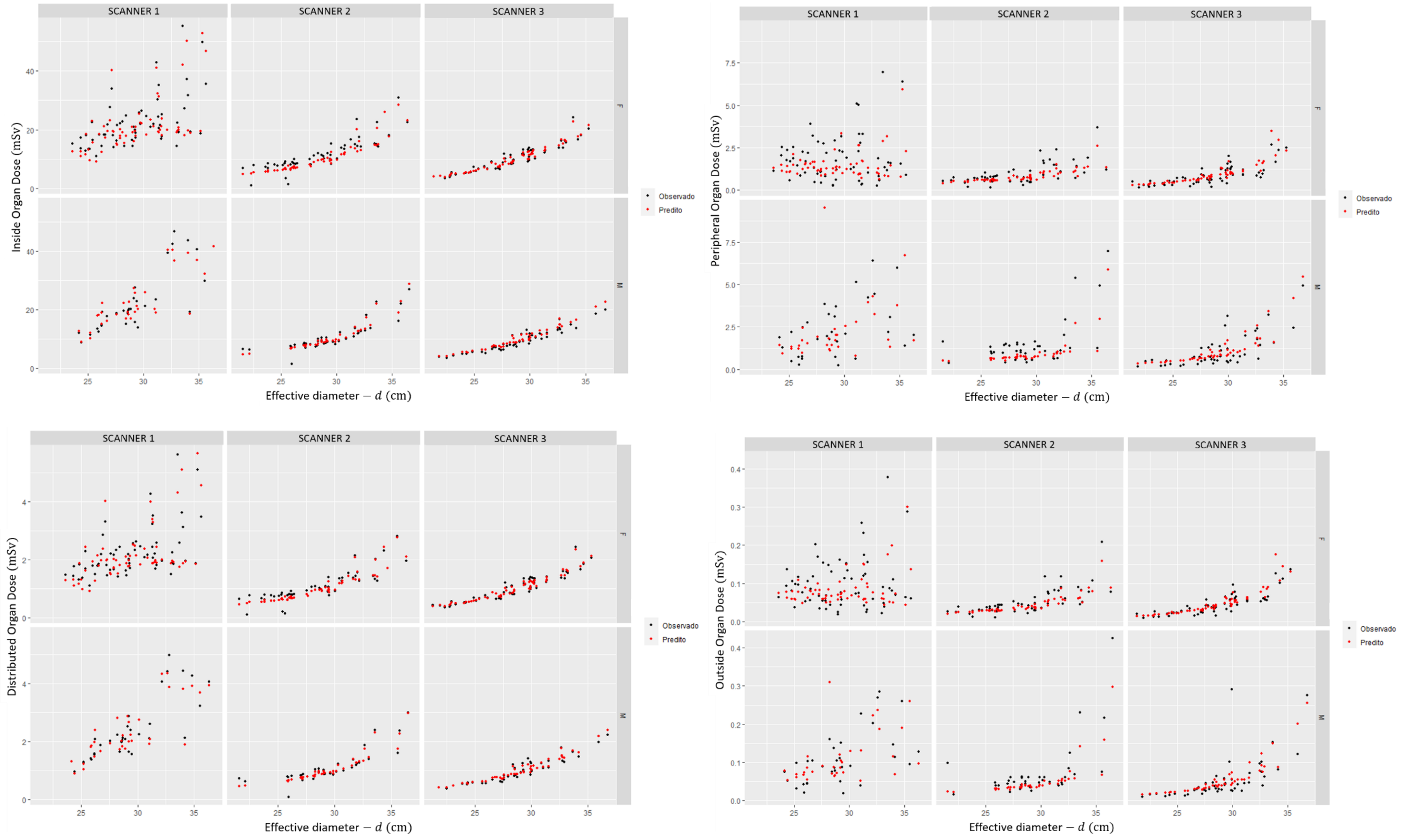

Figure S3. A) Inside Organ Dose; B) Peripheral Organ Dose; C) Distributed Organ Dose and D) Outside Organ Dose values for Abdomen-pelvis protocol of female (F) and male (M) patients computed using NCICT (original) and predicted from GAM as a function of effective diameter, d.

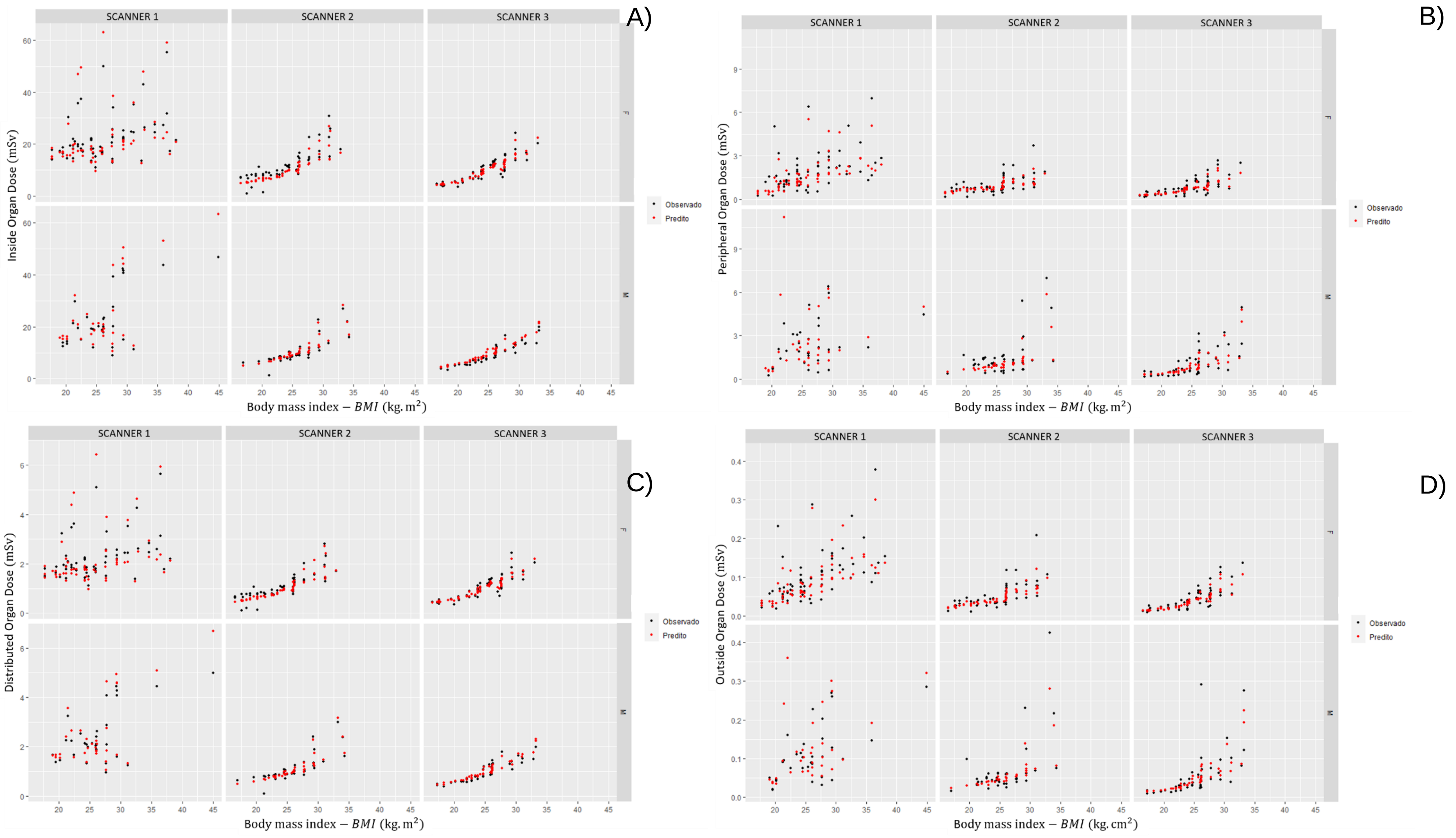

Figure S4. A) Inside Organ Dose; B) Peripheral Organ Dose; C) Distributed Organ Dose and D) Outside Organ Dose values for Abdomen-pelvis protocol of female (F) and male (M) patients computed using NCICT (original) and predicted from GAM as a function of body mass index, BMI.

Bibliography

1. Stasinopoulos DM, Rigby RA. Generalized Additive Models for Location Scale and Shape (GAMLSS) in R. Journal of Statistical Software 2007;23(7):1 - 46. doi: 10.18637/jss.v023.i07

2. Wood SN. Generalized Additive Models: An Introduction with R. Boca Raton: Chapman and Hall/CRC, 2017: 496.

3. Weisberg S. Applied linear regression: Wiley, 2014.
